# Development and Validation of a Combined Model for Preoperative Prediction of Lymph Node Metastasis in Peripheral Lung Adenocarcinoma

**DOI:** 10.3389/fonc.2021.675877

**Published:** 2021-05-24

**Authors:** Qi Li, Xiao-qun He, Xiao Fan, Chao-nan Zhu, Jun-wei Lv, Tian-you Luo

**Affiliations:** ^1^ Department of Radiology, The First Affiliated Hospital of Chongqing Medical University, Chongqing, China; ^2^ Department of Radiology, Children’s Hospital of Chongqing Medical University, National Clinical Research Center for Child Health and Disorders, Ministry of Education Key Laboratory of Child Development and Disorders, Chongqing, China; ^3^ Hangzhou YITU Healthcare Technology, Hangzhou, China

**Keywords:** radiomics, lymph node metastasis, computed tomography, lung adenocarcinoma, machine learning

## Abstract

**Background:**

Based on the “seed and soil” theory proposed by previous studies, we aimed to develop and validate a combined model of machine learning for predicting lymph node metastasis (LNM) in patients with peripheral lung adenocarcinoma (PLADC).

**Methods:**

Radiomics models were developed in a primary cohort of 390 patients (training cohort) with pathologically confirmed PLADC from January 2016 to August 2018. The patients were divided into the LNM (−) and LNM (+) groups. Thereafter, the patients were subdivided according to TNM stages N0, N1, N2, and N3. Radiomic features from unenhanced computed tomography (CT) were extracted. Radiomic signatures of the primary tumor (R1) and adjacent pleura (R2) were built as predictors of LNM. CT morphological features and clinical characteristics were compared between both groups. A combined model incorporating R1, R2, and CT morphological features, and clinical risk factors was developed by multivariate analysis. The combined model’s performance was assessed by receiver operating characteristic (ROC) curve. An internal validation cohort containing 166 consecutive patients from September 2018 to November 2019 was also assessed.

**Results:**

Thirty-one radiomic features of R1 and R2 were significant predictors of LNM (all P < 0.05). Sex, smoking history, tumor size, density, air bronchogram, spiculation, lobulation, necrosis, pleural effusion, and pleural involvement also differed significantly between the groups (all P < 0.05). R1, R2, tumor size, and spiculation in the combined model were independent risk factors for predicting LNM in patients with PLADC, with area under the ROC curves (AUCs) of 0.897 and 0.883 in the training and validation cohorts, respectively. The combined model identified N0, N1, N2, and N3, with AUCs ranging from 0.691–0.927 in the training cohort and 0.700–0.951 in the validation cohort, respectively, thereby indicating good performance.

**Conclusion:**

CT phenotypes of the primary tumor and adjacent pleura were significantly associated with LNM. A combined model incorporating radiomic signatures, CT morphological features, and clinical risk factors can assess LNM of patients with PLADC accurately and non-invasively.

## Introduction

Despite advances in early detection, diagnosis, staging, and treatment, lung cancer still remains the leading cause of death worldwide (1). Additionally, peripheral lung adenocarcinoma (PLADC), defined as adenocarcinoma occurring below segmental bronchus, is the most common histological subtype of lung cancer (2). Evaluating the status of lymph node metastasis (LNM) accurately is of great benefit to the treatment strategy decision and prognosis of patients with PLADC.

Previous studies (3, 4) have reported a significant association between LNM and computed tomography (CT) features and clinicopathological variables, including tumor centrality, consolidation-to-tumor ratio, age, papillary/micropapillary predominant subtype, and more advanced T stage in non-small cell lung cancer. Some researchers have reported that pleural involvement on preoperative CT images had a moderate correlation with visceral pleural invasion (5, 6). Chang et al. (7) concluded that lymphatic and visceral pleural surface invasion could be used to predict LNM. In other words, previous studies have concluded that pleural involvement was closely related to LNM (5–7). Therefore, we hypothesized that the primary tumor is a “seed,” adjacent pleura is the “soil,” and tumor cells could inseminate systematically through subpleural lymphatics owing to abundant lymphatic and vascular networks within the sub-pleura. Although previous studies have shown that several histological parameters can be predictors of LNM, these evaluation parameters are only available postoperatively. Preoperative knowledge of LNM can provide valuable information for determining the scope of surgical resection and the need of adjuvant therapy (8–10).

Radiomics, the high-throughput extraction of advanced quantitative imaging features from radiographic images, has attracted increased attention of physicians in recent years and has shown promise in characterizing tumor phenotypes, including imaging diagnosis, treatment, and prediction of prognosis and treatment efficacy of tumors (11–13). Recent studies have recognized the contribution of radiomics in the preoperative assessment of lymph node status in lung cancer (14–17). However, these studies predicted LNM of lung cancer mainly by extracting the quantitative information of the tumor itself. To the best of our knowledge, whether the combination of the radiomic signatures of the primary tumor (R1) and those of adjacent pleura (R2) can produce a superior prediction of LNM for patients with PLADC have not yet been established.

Therefore, the study aim was to develop and validate a combined model that incorporates R1, R2, and CT morphological features and identify clinical risk factors for predicting LNM in patients with PLADC.

## Methods

### Patient Selection

This study obtained ethical approval from the institutional review board in our hospital, and the need for informed consent was waived due to the retrospective nature of the study. A total of 390 patients with pathologically confirmed PLADC during January 2014 to August 2018 were included as a training cohort. **Data Supplement A1** presents the patient recruitment flowchart as well as the inclusion and exclusion criteria of this study.

Patients in the training cohort were divided into the LNM (+) group (n = 228) and LNM (−) group (n = 162), with an average age of 60.36 ± 9.86 years (range: 24–83 years). Additionally, all patients were subdivided into N0 (n = 162, no regional node metastasis), N1 (n = 56, metastasis in ipsilateral pulmonary or hilar nodes), N2 (n = 156, metastasis in ipsilateral mediastinal/subcarinal nodes), and N3 (n = 13, metastasis in contralateral mediastinal/hilar, or supraclavicular nodes) according to the 8th edition of the Tumor–Node–Metastasis (TNM) classification. Clinical characteristics, including age, sex, and smoking history, were collected. In addition, data from 166 consecutive patients with PLADC (N0 = 75, N1 = 19, N2 = 61, N3 = 11) with a mean age of 60.51 ± 9.19 years (range: 42–81 years) in our institution during September 2018 to November 2019 were collected and included as an internal validation cohort.

### CT Image Acquisition and Morphological Features Analysis

Chest CT scan was performed with Discovery 750 HD CT (GE Health care, Milwaukee, WI, USA), and the original images were reconstructed using a medium sharp reconstruction algorithm with a thickness of 0.625–1.25 mm and transmitted to the Picture Archiving and Communication System (PACS). CT features were reviewed in both lung window images (window width: 1600 Hounsfield units [HU]; window level: −600 HU) and mediastinal window images (window width: 400 HU; window level: 40 HU).

**Figure 1:**
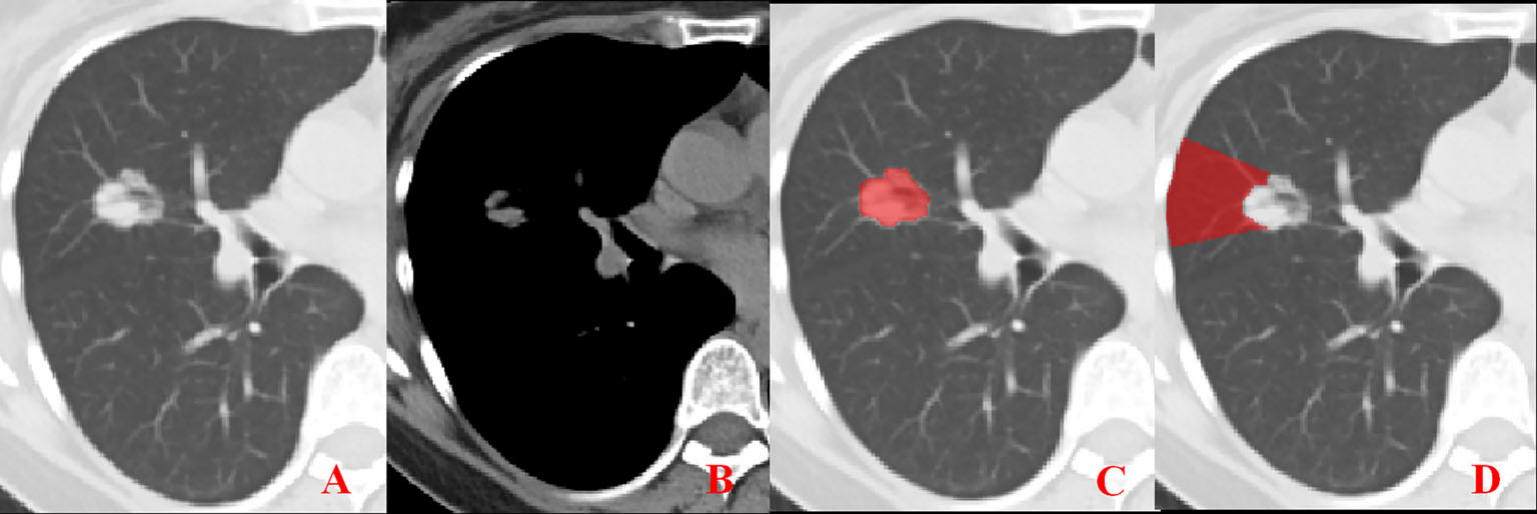
Representative image showing no pleural involvement. **(A, B)** No pleural involvement is seen in either the lung window or mediastinal window images. **(C, D)** ROI delineation of the primary tumor and nearby pleura.

**Figure 2:**
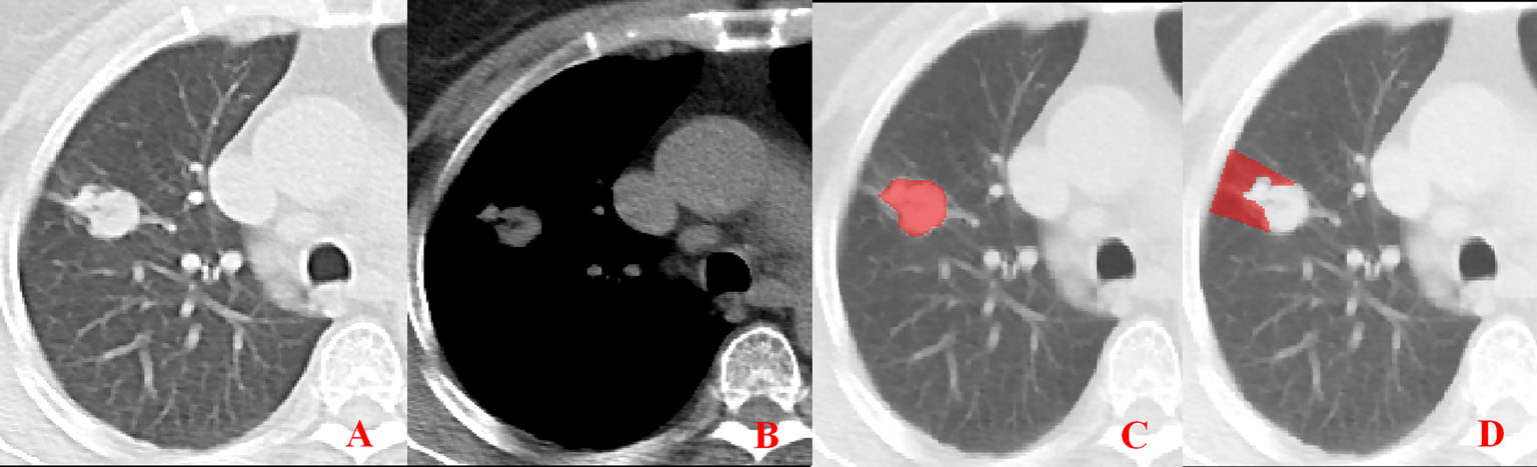
Pleural involvement of Type I. **(A, B)** One or more linear shadows are observed between the tumor and pleura in the lung window images but are not observed in the mediastinal window images. **(C, D)** ROI delineation of the primary tumor and nearby pleura.

**Figure 3:**
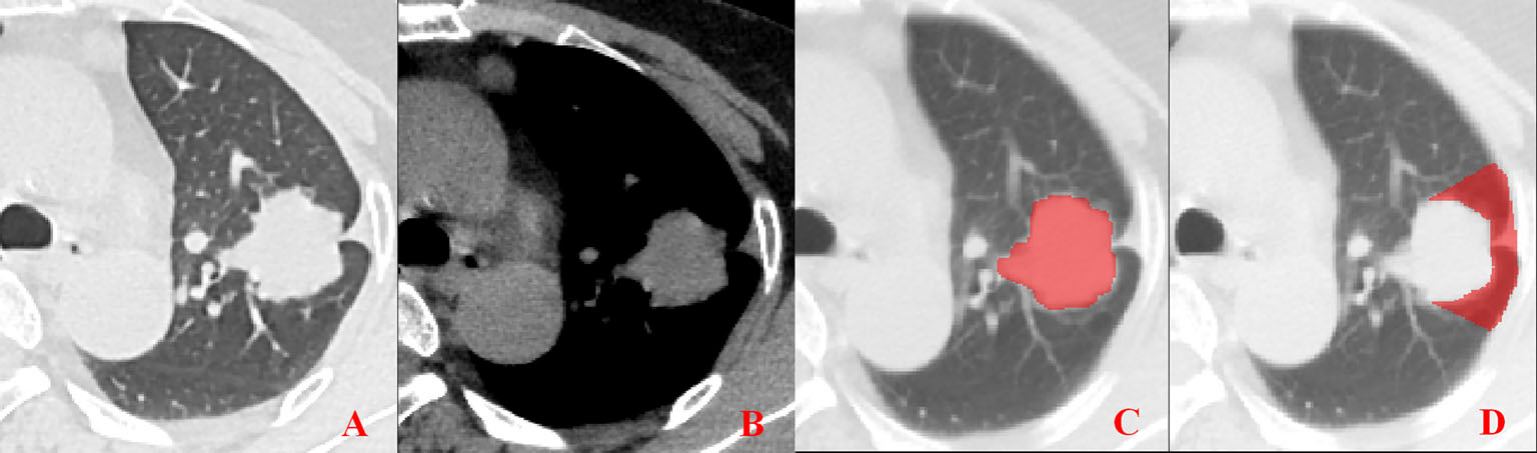
Pleural involvement of Type II. **(A, B)** Linear or cord-like shadows are observed between the tumor and pleura in both the lung window and mediastinal window images. **(C, D)** ROI delineation of the primary tumor and nearby pleura.

**Figure 4:**
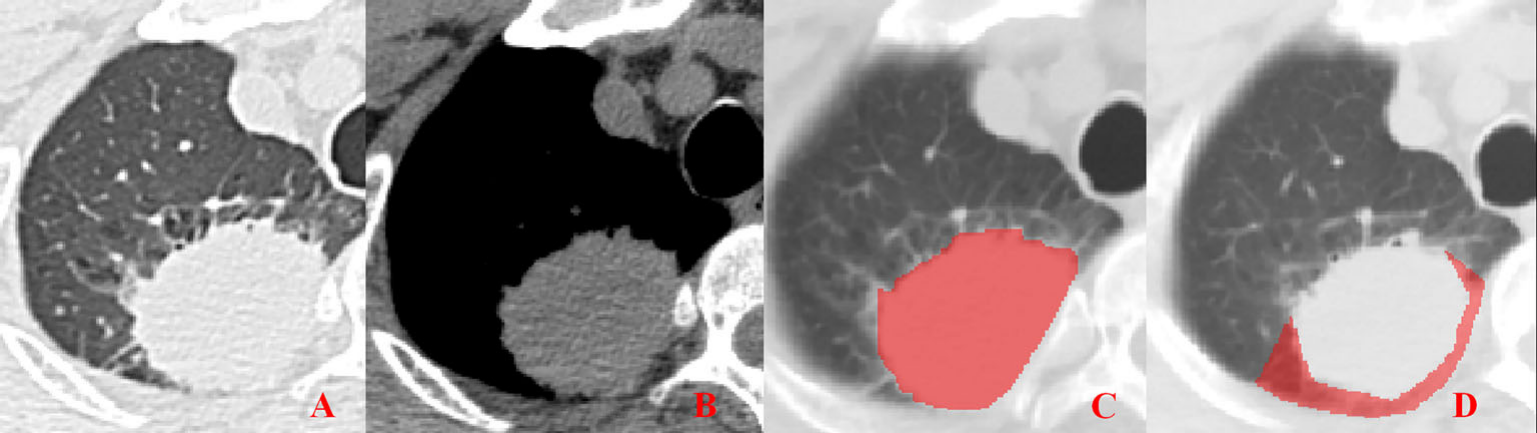
Pleural involvement of Type III. **(A, B)** Tumor attached to the pleura with a broad base observed in both the lung window and mediastinal window images. **(C, D)** ROI delineation of the primary tumor and nearby pleura.

A senior radiologist (with 18 years of work experience in thoracic imaging diagnosis) and a junior radiologist (with 13 years of work experience in thoracic imaging diagnosis) reviewed the CT images to reach a consensus. Tumor size (the longest diameter of the tumor on cross-sectional images), tumor density (solid or sub-solid), air space, air bronchogram, lobulation, spiculation, pleural effusion, necrosis, and pleural involvement were measured and evaluated. Referring to the standards established in previous research (3), pleural involvement was classified into three types (**Figures 1**–**4**): Type I, which manifested as one or more linear shadows between tumor and pleura on lung window images but was not observed on mediastinal window images; Type II, which manifested as linear or cord-like shadows between the tumor and pleura observed in both lung windows and mediastinal window images; and Type III, which were tumors attached to the pleura with a broad base. For tumors with concurrent Type I, Type II, or Type III presentation, the pleural involvement was recorded as the latter type.

### Radiomic Feature Selection and Signature Building

Unenhanced CT images of PLADC were extracted from PACS and then exported to the ITK-SNAP software (version 2.2.0, www.itk-snap.org) for manual segmentation. Considering that LNM depends on the synergies of the primary tumor and nearby pleura, both of them are investigated. For the primary tumor, the largest slice of tumor was selected from axial CT images, and regions of interest (ROIs) were carefully drawn on it and adjacent two slices, covering the whole contour of tumor. For all nearby pleura delineation, we tried to avoid the soft tissue and ribs of the chest wall; additionally, all pleural ROI delineation was defined as two lines tangent to the edges of the tumor, intersecting the visceral pleura at 90°. If there was no pleural involvement, ROI was drawn on the region between the primary tumor and pleura on the largest slice of tumor and adjacent two slices; if there was pleural involvement of Type I, Type II, and Type III, three adjacent slices showing the sign of pleural involvement most clearly were selected and delineated (**Figures 1**–**4**). To ensure consistency, these delineations were performed three times, and reproducibility assessment on intra-reader agreement were assessed by intraclass correlation coefficients (ICCs) for radiomics feature extraction after ROI delineation, ICC > 0.75 were retained as they showed good agreement between different segmentations.

Radiomic feature extraction was performed on PyRadiomic platform implemented in Python (https://pyradiomics.readthedocs.io/en/latest/), which can extract radiomic features from CT images *via* an algorithm with a large panel of engineered hard-coded features, such as morphological features (ROI size, volume, surface area, etc.), first-order features (geometric morphology and histogram features), second-order texture features (gray level co-occurrence matrix, gray level long matrix, gray level generation matrix, and neighborhood gray difference matrix), and other features based on filtering and transformation (wavelet transform).

As shown in **Supplementary Figure A2**, radiomic feature selection and signature building of R1 and R2, including these steps, were performed. First, we normalized the resolution feature matrix. For each vector, we calculated the L2 norm and divided it. The feature vector was then mapped to a unit vector. Second, we compared the similarity of each feature pair due to the high dimensionality of the radiomic features space. If the Pearson correlation coefficient of a feature pair was greater than 0.90, we randomly removed one feature pair. Third, we combined the optimal subset method with a minimum Akaike’s Information Criterion (AIC) to select the best combination of features. The optimal subset method can provide the corresponding χ^2^ value in the case where all feature number combinations are different, but it cannot identify the best combination. Therefore, the corresponding AIC values under various combinations could be calculated to find the smallest corresponding AIC value. We built a final logistic regression model using a combination of features under the minimum AIC correspondence. Using this method, we selected features to build the R1 and R2 models. Finally, after traversing five machine-learning algorithms, we chose multinomial logistic regression as the final classifier.

### Radiomics Model Construction and Evaluation

R1 and R2 models that reflected the radiomics signature of the primary tumor and adjacent pleura were established; an R1+R2 model was also constructed as a whole ROI to explore the ability to predict LNM in patients with PLADC. A combined model, including R1 and R2, CT morphological features, and clinical risk factors, was developed by multivariate logistic regression analysis. Moreover, a combined nomogram based on the logistic regression model was then plotted. Hosmer–Lemeshow goodness of fit test was applied to evaluate the calibration of the combined model, and the results were represented by a calibration curve.

### Lymph Node Status Ascertainment

All patients underwent lobectomy or a more extensive resection. Systematic lymph node dissection was performed in all patients according to the European Society of Thoracic Surgeons guidelines (18, 19). The minimal number of dissected lymph nodes was six and at least three mediastinal lymph nodal stations and subcarinal stations had to be included. The hilar and intrapulmonary lymph nodes were excised as well. All surgical specimens and lymph nodes were fixed in 10% formalin and then sliced at the maximum dimension, and all sections were embedded in paraffin. Two experienced pathologists blindly evaluated all slices and lymph nodes together, and any disagreement was resolved by consensus. Pathological TNM stage, histological type, and lymph node station were evaluated according to the 8th edition of the TNM classification of lung cancer (2017) provided by the International Union against Cancer and the American Joint Commission on Cancer (20, 21).

### Statistical Analysis

Statistical analysis was performed using SPSS statistics (version 24; IBM, Armonk, NY, USA) and R software (version3.6.1; http://www.Rproject.org). For continuous variables of clinical characteristics and CT morphological features, independent t-test or Mann–Whitney U test was performed; for categorical variables, Chi-square test was used for comparisons between the two groups. The combined model was constructed with multivariate logistic regression analysis and the performance of the combined model was evaluated using receiver operating characteristic (ROC) curve. A combined nomogram and a calibration curve of the combined model were then plotted. A calibration curve showing discrete experimental points close to or nearly coinciding with the diagonal would indicate that the calibration of the combined model was high. A two-sided *P* value < 0.05 was considered to be indicative of statistical significance.

## Results

### Clinical Characteristics and CT Morphological Features

Males (*P* = 0.025) and smokers (*P* = 0.005) were more common in the LNM (+) group than in the LNM (−) group. However, no significant difference in age was observed between the two groups (*P* = 0.794). Tumor size, density, air bronchogram, spiculation, lobulation, necrosis, pleural effusion, and pleural involvement were found to be associated with LNM (all *P* < 0.05). Tumor size was larger in the LNM (+) group than that in the LNM (−) group (*P* < 0.001). Tumors with solid density, air bronchogram, spiculation, lobulation, necrosis, and pleural effusion were more common in the LNM (+) group than in the LNM (−) (all *P* < 0.05). However, there were no significant differences in air space and vascular convergence between the two groups (all *P* > 0.05, **Table 1**).

**Table 1:** Comparison of the clinical characteristics and CT morphological features between the LNM (−) and LNM (+) groups (n, %).

Characteristics	LNM(−) group (237)	LNM(+) group (319)	Sig.	*P* value
Age (years)	60.28 ± 10.21	60.50 ± 9.33	0.261	0.794^a^
Sex (male)	111 (46.84%)	180 (56.43%)	5.014	0.025^b^
Smoker	82 (34.60%)	147 (46.08%)	7.931	0.005^b^
Tumor size (mm)	23.00 (16.00, 30.00)	32.00 (24.00, 42.00)	9.023	< 0.001^c^
Density			82.686	< 0.001^b^
Solid	162 (68.35%)	308 (96.55%)		
Sub-solid	75 (31.65%)	11 (3.45%)		
Air space	74 (31.22%)	81 (25.39%)	2.300	0.129^b^
Air bronchogram	53 (22.36%)	32 (10.03%)	15.966	< 0.001^b^
Spiculation	56 (23.63%)	134 (42.01%)	20.415	< 0.001^b^
Lobulation	209 (88.19%)	304 (95.30%)	9.639	0.002^b^
Necrosis	20 (8.44%)	64 (20.06%)	14.325	< 0.001^b^
Vascular convergence	54 (22.78%)	69 (21.63%)	0.105	0.746^b^
Pleural effusion	2 (0.84%)	13 (4.08%)	5.409	0.020^b^
Pleural involvement			44.470	< 0.001^b^
Absent	23 (9.70%)	20 (6.27%)		*P^#^*
Type I	144 (60.76%)	114 (35.74%)		*P* ^*^
Type II	30 (12.66%)	82 (25.71%)		*P* ^*^
Type III	40 (16.88%)	103 (32.29%)		*P* ^*^

^a^independent t-test; ^b^Chi-squared test; ^c^Mann–Whitney U test; P^#^ means P > 0.05 and P^*^ means P < 0.05 for further pairwise comparison between two groups. LNM, lymph node metastasis; CT, computed tomography.

### Radiomics Model Construction

**Figure 5:**
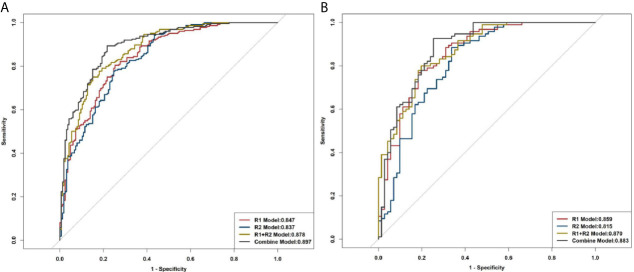
ROC curves of R1, R2, R1+R2, and the combined model for distinguishing LNM. **(A)** Training cohort. **(B)** Validation cohort.

The R1 model was built with 13 features, including original first-order variance, wavelet transform, gray histogram features, gradient, and lbp.3D.k glszm small-area emphasis; the areas under the ROC curves (AUCs) for predicting LNM were 0.847 and 0.859 in training cohort and validation cohort, respectively (**Figure 5**). The R2 model was built with 19 features, including wavelet, square root, logarithm, and gradient, with AUCs of 0.837 and 0.815 for the prediction of LNM in the training cohort and validation cohort, respectively. In total, 1300 features were extracted from both the primary tumor and pleura. After ranking these features, 31 features from R1 and R2 were found to be significantly associated with LNM (all *P* < 0.05), and AUCs of R1+R2 model were 0.878 and 0.870 in the training and validation cohorts, respectively (**Figure 5**). Furthermore, the combined model was also developed with AUCs of 0.897 and 0.883 for the training and validation cohorts, respectively (**Figure 5**).

### Evaluation of the Radiomics Models

**Figure 6:**
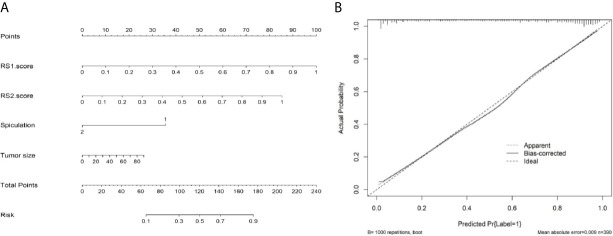
Nomogram and calibration curve of radiomic models. **(A)** Nomogram of the combined model. **(B)** Calibration curve showing that the discrete experimental points are coincident with the diagonal, which indicates that the calibration of the combined model is high.

Multivariable analysis revealed that long diameter, presence of spiculation, radiomics score of the primary tumor (RS1), and radiomics score of the pleura around the tumor (RS2) were significant predictors (**Table 2**). Therefore, they were fused as a radiomics nomogram (**Figure 6A**). The calibration curve showed that the discrete experimental points were similar to or the same as the diagonal, which indicated that the calibration of the combined model was high (**Figure 6B**).

**Table 2:** Variables and coefficients of the radiomics nomogram.

Variables	*β*	Adjusted OR (95% CI)	*P* value
RS1 (per 0.1 increase)	3.9867	53.88 (14.89–215.1)	< 0.0001
RS2 (per 0.1 increase)	3.4074	30.19 (8.73–112.25)	< 0.0001
Tumor size	0.0117	1.01 (0.99–1.04)	0.3856
Spiculation	−1.4176	0.24 (0.13–0.45)	< 0.0001
Intercept	−1.9709	0.14 (0.04–0.44)	0.0009

RS1, radiomics score of the primary tumor; RS2, radiomics score of the adjacent pleura.

### Radiomics Model for Identifying N0, N1, N2, and N3

**Figure 7:**
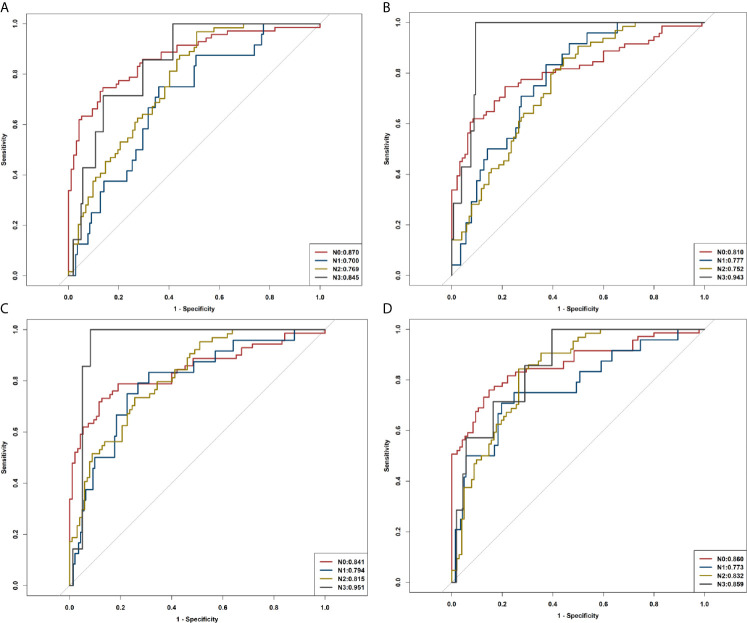
ROC curves of R1, R2, R1+R2, and the combined model in identifying N0, N1, N2, and N3. **(A)** ROC curves of R1. **(B)** ROC curves of R2. **(C)** ROC curves of R1+R2. **(D)** ROC curves of the combined model.

Radiomic signatures also showed good performance in identifying the lymph node stage of N0, N1, N2, and N3 (**Supplementary Figure A3**) as shown by the following AUCs: for the R1 model, 0.839, 0.691, 0.768, and 0.864 in the training cohort and 0.870, 0.700, 0.769, and 0.845 in the validation cohort, respectively; for the R2 model, 0.808, 0.783, 0.763, and 0.885 in the training cohort, and 0.810, 0.777, 0.752, and 0.943 in the validation cohort, respectively; for the R1+R2 model, 0.866, 0.812, 0.824, and 0.927 in the training cohort and 0.841, 0.794, 0.815, and 0.951 in the validation cohort, respectively; and for the combined model, 0.916, 0.797, 0.823, and 0.927 in the training cohort and 0.860, 0.773, 0.832, and 0.859 in the validation cohort, respectively (**Table 3**, **Figure 7**).

**Table 3:** AUCs of radiomics models for evaluating lymph node staging.

Models	Training cohort				Validation cohort			
	N0	N1	N2	N3	N0	N1	N2	N3
R1	0.839	0.691	0.768	0.864	0.870	0.700	0.769	0.845
R2	0.808	0.783	0.763	0.885	0.810	0.777	0.752	0.943
R1+R2	0.866	0.812	0.824	0.927	0.841	0.794	0.815	0.951
Combined model	0.916	0.797	0.823	0.927	0.860	0.773	0.832	0.859

N0, No regional node metastasis; N1, Metastasis in ipsilateral pulmonary or hilar nodes; N2, Metastasis in ipsilateral mediastinal/subcarinal nodes; N3, Metastasis in contralateral mediastinal/hilar or supraclavicular nodes.

## Discussion

Radiologic examinations, including CT, magnetic resonance imaging (MRI), and positron emission tomography combined with CT (FDG-PET/CT), can be used for pretherapeutic lymph node assessments (22–24). As an alternative, CT is an important part of the PLADC staging process in clinical practice. However, some previous studies have observed low sensitivity and specificity of CT, and others have shown that CT was severely limited when relying solely on a short-axis diameter of ≤10 mm of the thoracic lymph nodes in accurately evaluating malignant nodes (25, 26). Diffusion-weighted magnetic resonance imaging (DWI) of MRI has been applied in lung cancer staging for the last two decades; however, further development of protocols and more clinical trials for lymph node evaluation are still needed (23). FDG-PET/CT has been reported to be superior to CT for evaluating LNM of lung cancer, but high false-positive rate and radiation dosage have restricted its clinical application (27). Therefore, preoperative imaging for noninvasive evaluation of the status of lymph nodes is highly desirable. In the present study, we developed and validated a radiomics signature-based model that incorporates radiomic signatures of both the primary tumor and adjacent pleura, CT morphological features, and clinical factors for prediction of LNM in patients with PLADC.

In this study, R1, which reflects radiomic signatures of the primary tumor had AUCs of 0.847 and 0.859 for predicting LNM in the training and validation cohorts, respectively, suggesting a huge potential for radiomics in predicting LNM. Consistent with our results, previous researchers have also reported that radiomic signatures were of great value in predicting LNM in lung cancer (15, 28); Wang et al. (17) confirmed that radiomic signatures from peritumoral lung parenchyma would increase the prediction efficiency of LNM in clinical stage T1 lung adenocarcinoma. Additionally, R2, which showed radiomic signatures of pleura around the tumor, was associated with LNM in patients with PLADC, and yielded AUCs of 0.837 and 0.815 for predicting LNM in the training and validation cohorts, respectively. To the best of our knowledge, few studies have applied radiomic signatures of pleura around the tumor to predict LNM. Researchers have concluded that LNM depends on selected cancer cells (the “seeds”) and micro-environments (the “soil”), and metastases formed only when the seeds and soil were compatible (29, 30). We thus hypothesized the “seed and soil” theory for LNM prediction. Based on the “seed and soil” theory, interestingly, we found that LNM was associated with both the tumor and the phenotype of its nearby pleura. This finding might partly be explained by the rich subpleural lymph drainage and direct drainage route into the mediastinum, through which tumor cells may spread and metastasize easily (6, 31). We concluded that tumor invasion to the network of subpleural lymph vessel would lead to higher occurrence of LNM. Moreover, radiomic signatures of R1+R2, which contained 31 characteristics in total, showed good performance in predicting LNM in patients with PLADC, with AUCs of 0.878 and 0.870 in the training and validation cohorts, respectively.

Previous studies have confirmed that several CT features and clinical risk factors were closely related to LNM of lung adenocarcinoma (8, 32–39). Similarly, we found that sex, smoking history, and eight CT morphological features of tumors, including long diameter, tumor density, air bronchogram, spiculation, lobulation, necrosis, pleural effusion, and pleural involvement, were significantly associated with LNM in this study. Therefore, we further established a prediction model that combined radiomic signatures of R1 and R2, CT features, and clinical risk factors. The combined model is of great value in predicting LNM with AUCs of 0.897 and 0.883 in the training and validation cohorts, respectively. The decision curve showed that the combined model was of great help in clinical decision-making. We have also developed a radiomics nomogram and calibration curve of the combined model, both of which showed that the combined model had good predictive ability for LNM in patients with PLADC.

Asamura et al. (40) reported that the 5-year survival rates in patients with lung cancer according to the pathological N statuses were 75% (N0), 49% (N1), 36% (N2), and 20% (N3). Therefore, the survival differed significantly between all neighboring nodal categories, and it is very important to accurately evaluate the metastasis status of lymph nodes before operation. In the present study, the radiomics model was also used to distinguish N0, N1, N2, and N3, and the combined model revealed good diagnostic performance in estimating N stages for patients with PLADC.

The present study had several limitations. All data were collected within a single institution, but we are preparing to conduct a multicenter study to verify the reliability and general applicability of this model. Previous studies have shown the relationship between different pleural involvement and LNM or nodal staging. Radiomics was used only to further quantify the relevant features, and we believe that we can achieve good performance in external verification. Moreover, due to the lack of MRI and PET images, there is scope for improving the performance of the model, especially under the condition wherein PET/CT can provide better reference for evaluating LNM. We chose only three slices instead of the whole tumor for image-feature extraction. Future work might benefit from automatic target area delineation software, and more auxiliary information around the tumor can be added to achieve an accurate assessment of tumor lymph nodes.

## Conclusion

This study showed that obtaining information about the primary tumor and pleura around the tumor provides complementary information that can be useful in clinical decision-making. The combined model, which incorporates radiomic signatures, CT features, and clinical factors, can be used as an auxiliary tool to predict LNM in patients with PLADC.

## Data Availability Statement

The original contributions presented in the study are included in the article/**Supplementary Material**. Further inquiries can be directed to the corresponding author.

## Ethics Statement

The studies involving human participants were reviewed and approved by Ethics Review Committee of the First Affiliated Hospital of Chongqing Medical University. The ethics committee waived the requirement of written informed consent for participation.

## Author Contributions

QL and X-qH have contributed equally to this work and share first authorship. J-wL and T-yL contributed equally to this work and share correspondence authorship. All authors contributed to the article and approved the submitted version.

## Funding

This research was supported by the Science and Technology Innovation Program of Social Undertakings and People's Livelihood Security of Chongqing Science and Technology Commission to T-yL: Technology Exploration and Application of Precision Radiotherapy for Disease. (cstc2016shms-ztzx10002).

## Conflict of Interest

The authors declare that the research was conducted in the absence of any commercial or financial relationships that could be construed as a potential conflict of interest.

## Abbreviations

LNM, Lymph node metastasis; PLADC, Peripheral lung adenocarcinoma; CT, Computed tomography; R1, Radiomic signature of the primary tumor; R2, Radiomic signature of the adjacent pleura; TNM, Tumor–Node–Metastasis; N0, No regional node metastasis; N1, Metastasis in ipsilateral pulmonary or hilar nodes; N2, Metastasis in ipsilateral mediastinal/subcarinal nodes; N3, Metastasis in contralateral mediastinal/hilar, or supraclavicular nodes; PACS, Picture Archiving and Communication System; HU, Hounsfield units; ROIs, Regions of interest; ICCs, Intraclass correlation coefficients; AIC, Akaike information criterion; ROC, Receiver operating characteristic; AUCs, Area under the ROC curves; RS1, Radiomics score of the primary tumor; RS2, Radiomics score of the adjacent pleura; MRI, Magnetic resonance imaging; FDG-PET/CT, Fluorodeoxyribose positron emission tomography combined with CT; DWI, Diffusion-weighted magnetic resonance imaging.
