# Supplementary material for: Development and Validation of a Combined Model for Preoperative Prediction of Lymph Node Metastasis in Peripheral Lung Adenocarcinoma
Source: Front Oncol. 2021 May 24;11:675877. doi: 10.3389/fonc.2021.675877 (PMC8180898; doi:10.3389/fonc.2021.675877)

**Appendices**

Supplement Figure A1. Flowchart of patient enrollment, eligibility, and exclusion criteria of the datasets

Potentially eligible patients (n = 880)

- adenocarcinoma with definite lymph node status

- tumor located peripheral on CT images

- CT images within 1 month before surgery are available

Validation cohort (n = 166)

September 2018 - November 2019

Training cohort (n = 390)

January 2016 - August 2018

Data following eligibility inclusion and exclusion criteria (n = 556)

Excluded (n = 324)

- tumor with central location in CT images (128)

- with a history of anti-tumor before surgery (115)

- interval between CT and surgery of over 1 month (81)

Supplement Figure A2. Radiomics workflow in this study.


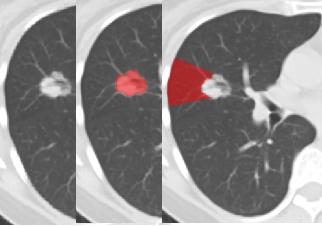


ROI Segmentation

Feature Selection:

Pearsom correction; Optimal subset; Minimum Akaike’s information criterion; Typical features

Feature Extraction:

Intensity; local variance; Binary; Gray level co-occurrence matrix; Morphology


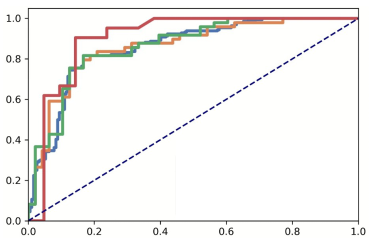

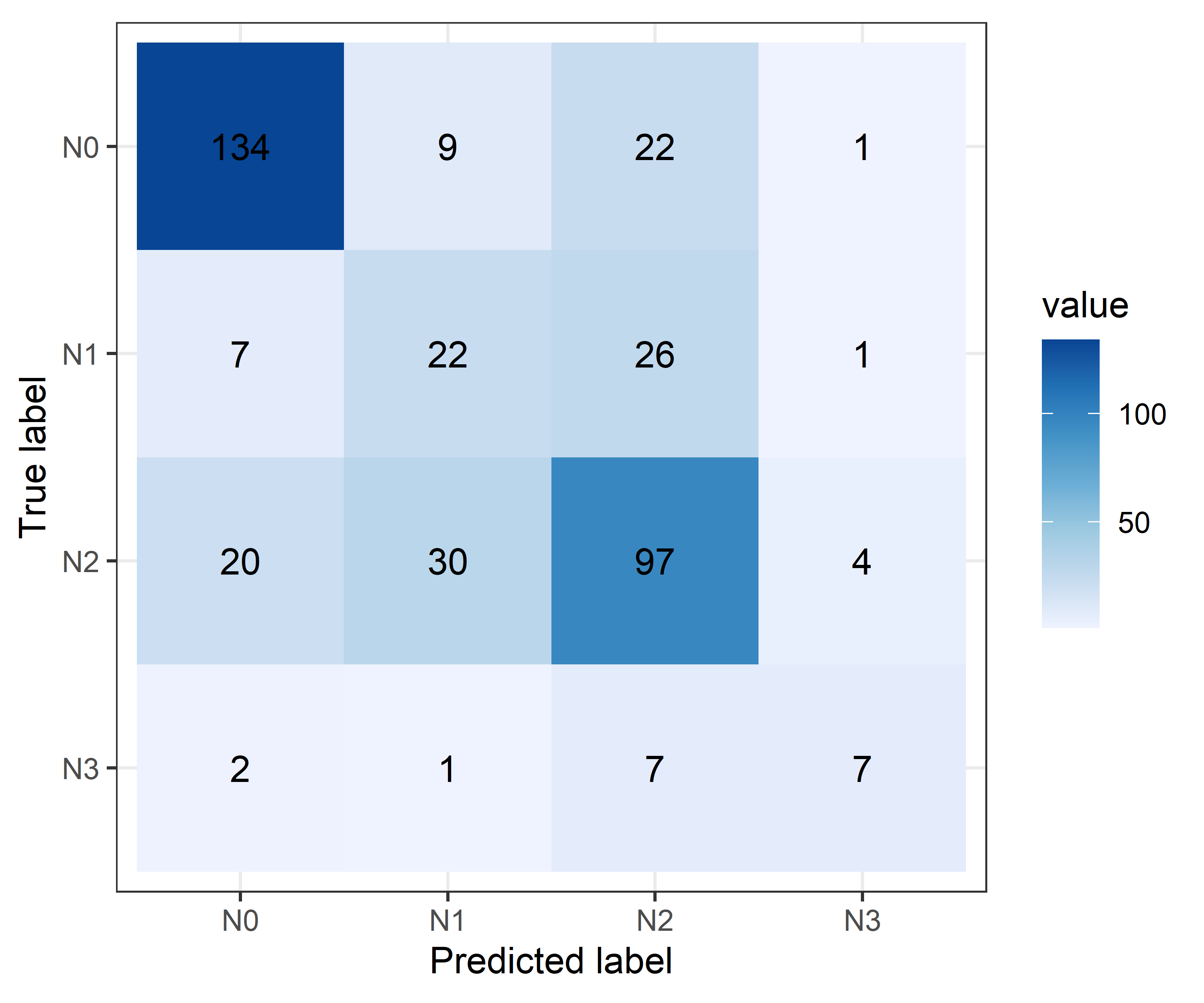


Machine learning:

K-nearest neighbor; Decision trees; Support vector machine; Multivariate logistic regression

Supplement Figure A3. The box-plot of R1, R2, R1+R2, and combined model in identifying N0, N1, N2, and N3 .


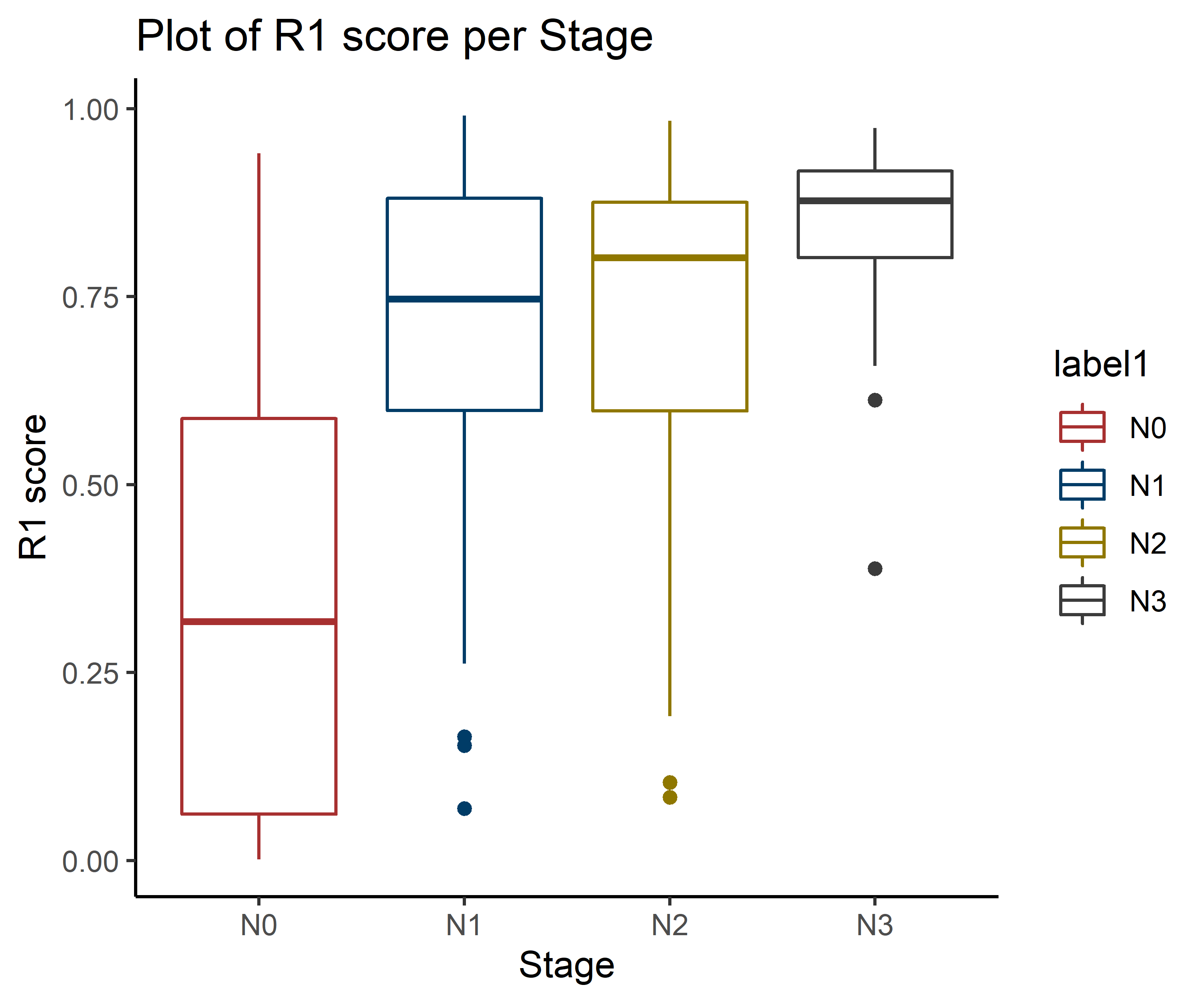

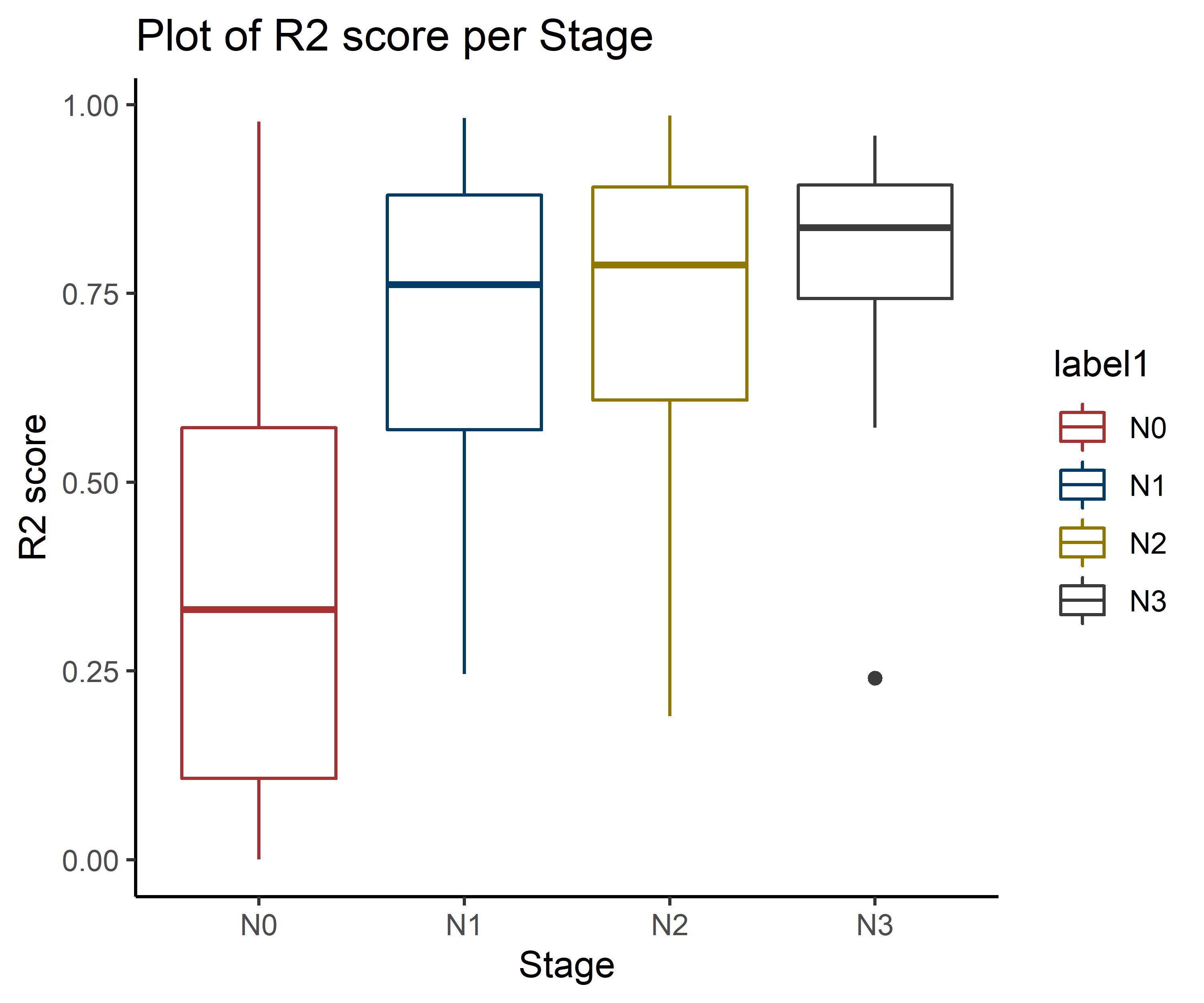


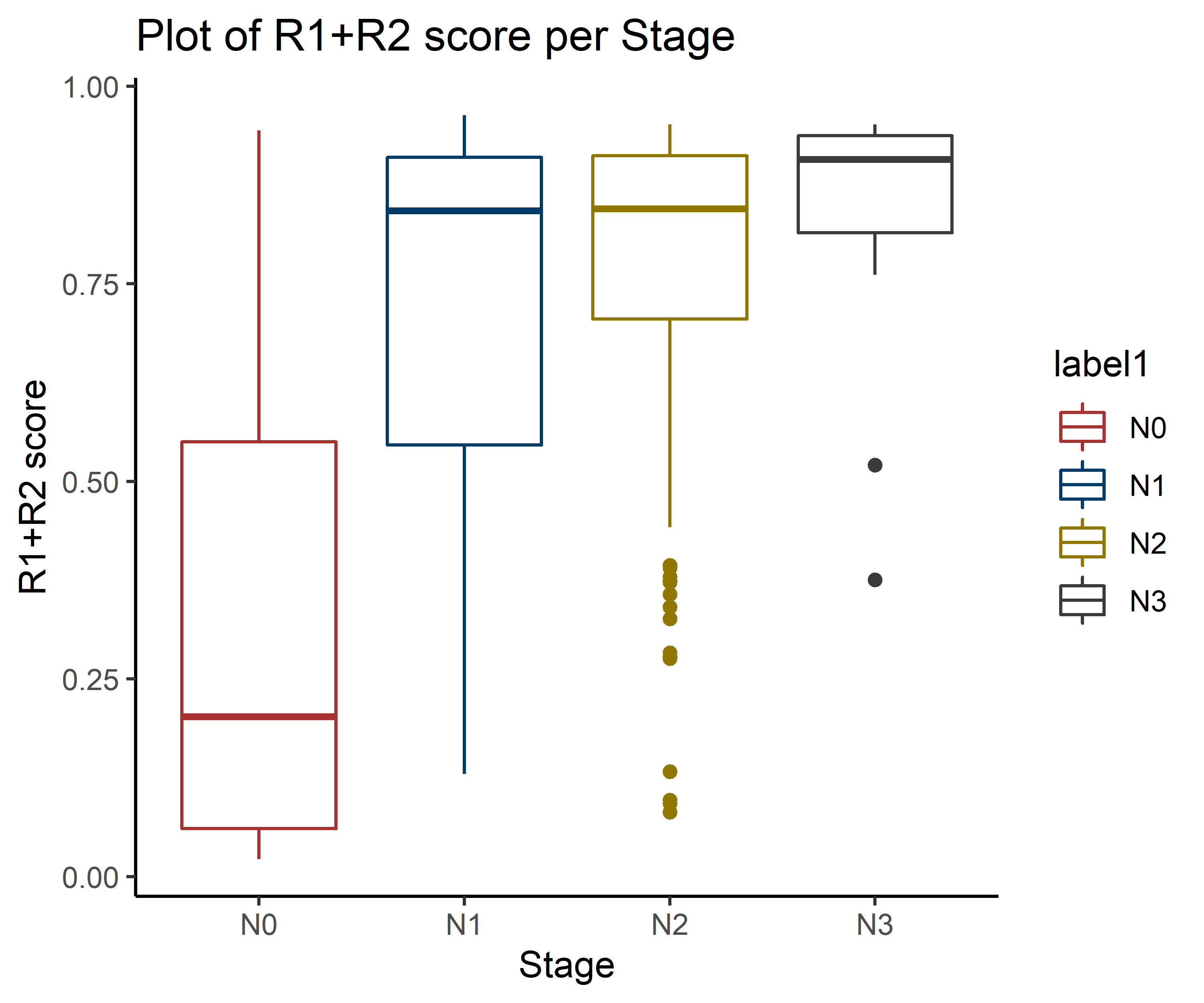

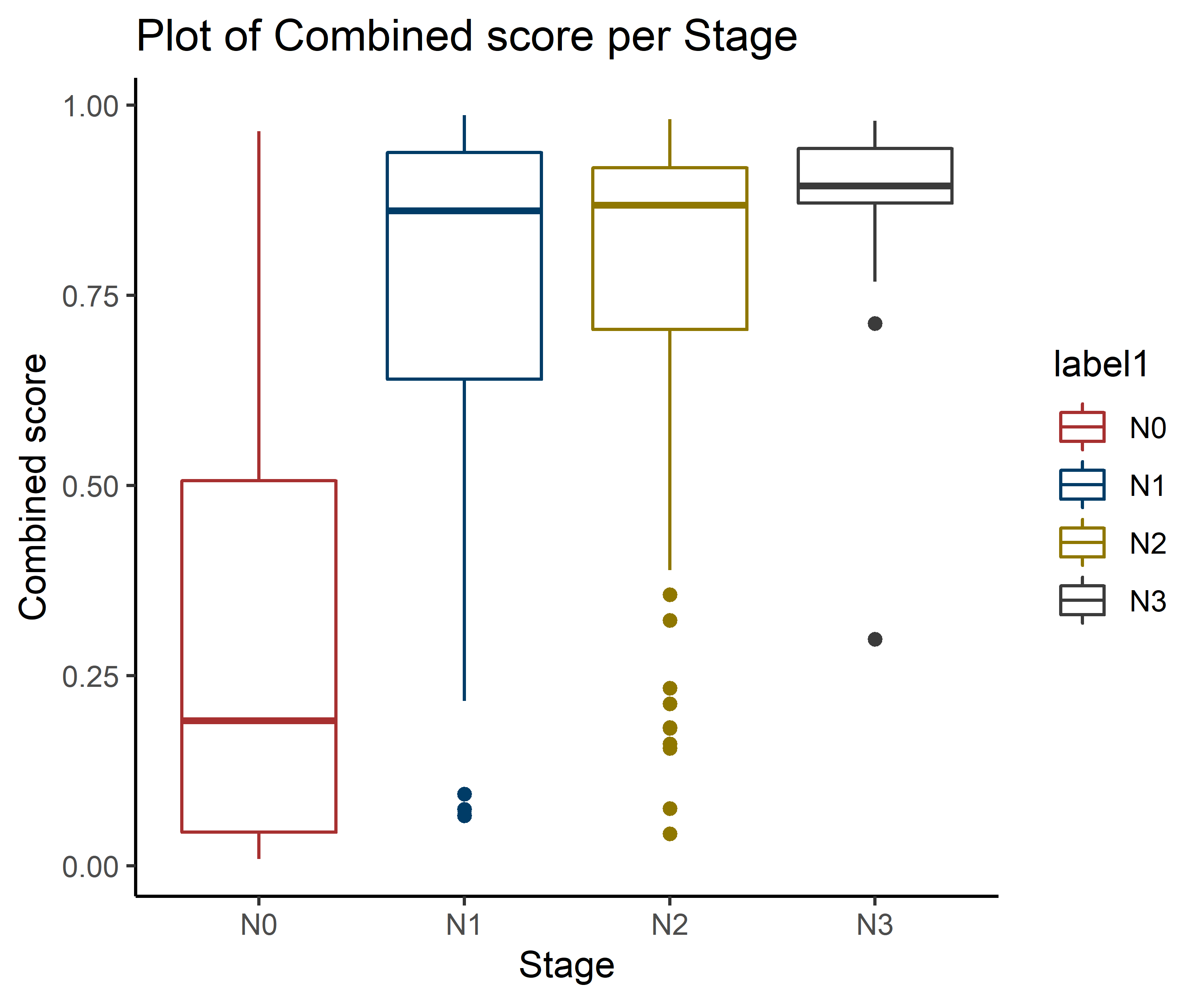

Supplement: Supplementary file 1 [file DataSheet_1.docx]
